# Thoughts on African Swine Fever Vaccines

**DOI:** 10.3390/v13050943

**Published:** 2021-05-20

**Authors:** Daniel L. Rock

**Affiliations:** Department of Pathobiology, College of Veterinary Medicine, University of Illinois at Urbana-Champaign, Urbana, IL 61802, USA; dlrock@illinois.edu

**Keywords:** Africa swine fever, ASF, vaccine, protective immunity, virulence and host range genes

## Abstract

African swine fever (ASF) is an acute viral hemorrhagic disease of domestic swine with mortality rates approaching 100%. Devastating ASF outbreaks and continuing epidemics starting in the Caucasus region and now in the Russian Federation, Europe, China, and other parts of Southeast Asia (2007 to date) highlight its significance. ASF strain Georgia-07 and its derivatives are now endemic in extensive regions of Europe and Asia and are “out of Africa” forever, a situation that poses a grave if not an existential threat to the swine industry worldwide. While our current concern is Georgia-07, other emerging ASFV strains will threaten for the indefinite future. Economic analysis indicates that an ASF outbreak in the U.S. would result in approximately $15 billion USD in losses, assuming the disease is rapidly controlled and the U.S. is able to reenter export markets within two years. ASF’s potential to spread and become endemic in new regions, its rapid and efficient transmission among pigs, and the relative stability of the causative agent ASF virus (ASFV) in the environment all provide significant challenges for disease control. Effective and robust methods, including vaccines for ASF response and recovery, are needed immediately.

“If you don’t know where you are going and you’ll end up someplace else”

Yogi Berra—New York Yankees baseball player.

## 1. Introduction

ASFV, the sole member of the Asfarviridae (Asfar, African swine fever, and related viruses), is a large, enveloped genetically complex virus containing a double-stranded DNA genome of approximately 190 kilobase pairs (kbp), which encodes over 170 proteins. Aspects of genome structure and replication strategy are shared between ASFV and other large dsDNA viruses, most notably the poxviruses [1,2].

ASFV is the only known DNA arbovirus. In sub-Saharan Africa, ASFV is maintained in a sylvatic cycle between wild swine (warthogs and bushpigs) and argasid ticks of the genus *Ornithodoros*. Unlike domestic swine, wild swine infected with ASFV are generally asymptomatic, with low viremia titers. Most adult warthogs in ASFV enzootic areas are seropositive and persistently infected. ASFV persistently infects ticks of the *Ornithodoros* spp. from which ASFV can be isolated years post-infection [1].

ASF occurs in several forms in domestic pigs, ranging from highly lethal (100% mortality) to subclinical. Hemostatic and hemodynamic changes (hemorrhage, edema, ascites, and shock) resulting from intravascular activation of coagulation are observed in pigs infected with highly virulent ASFV strains [3,4]. ASFV infects cells of the mononuclear-phagocytic system, including highly differentiated fixed-tissue macrophages and specific lineages of reticular cells, and highly virulent strains induce extensive damage in affected tissues [5,6,7]. The ability of ASFV to replicate and induce marked cytopathology in these cell types in vivo appears to be a critical factor in ASFV virulence. Viral and host factors responsible for the differing outcomes of ASFV infection remain poorly understood.

Notably, and of potential significance from a vaccine design and a disease control perspective, ASFV appears to establish long-term persistent/latent infections in warthogs and in domestic pigs surviving acute viral infection [8,9,10,11]. Under experimental conditions, the virus persists in a high percentage of domestic pigs infected with moderately virulent ASFV stains and is shed into the environment for at least 70 days post-infection [12]. In addition, viral DNA can be PCR-amplified from peripheral blood monocytes of pigs persistently infected with ASFV for at least 500 days post-infection [13]. Although the actual significance of ASFV persistent infection for virus perpetuation and transmission within domestic pig populations remains unclear [14], the detection of ASFV DNA in blood samples collected from clinically normal pigs at slaughter in an ASF endemic region suggests that chronically or persistently infected animals may be responsible for ASF persistence within endemic regions [15,16].

Despite the continual ASF threat and the fact that the disease was first described by Montgomery in 1921 [17], it is surprising that no ASF vaccine is available. Current data indicate that ASF vaccines could indeed be developed, as pigs surviving acute ASFV infection develop long-term resistance to homologous virus challenge but rarely to heterologous virus challenge [10,18,19,20,21,22]. Vaccine development has been hindered by large gaps in knowledge concerning aspects of ASFV infection and immunity, the extent of ASFV strain variation, and the ASFV proteins (protective antigens; (PA)) responsible for inducing protective immune responses in the pig.

## 2. Where We Are

### 2.1. ASF Protective Immunity

ASFV protective immunity remains poorly defined. As is the case with most viral infections, innate immunity and both humoral and cellular immune responses appear to be important for protection. Passive transfer of ASFV antibodies alone is sufficient to protect pigs from lethal ASFV infection [23,24,25]. The effector mechanisms associated with and the viral proteins inducing these antibody-mediated protective responses are undefined. ASFV neutralizing antibodies have been described [26,27,28,29,30,31], but they do not cross neutralize in vitro in a manner correlating with ASFV cross-protection in pigs–raising doubts as to their significance for protective immunity [27,32].

In vitro cytolytic effector functions mediated by anti-ASFV antibody have been described; however, no significant correlation has been found between complement-dependent antibody lysis or antibody-dependent cell-mediated cytotoxicity antibody titers in vitro and protective immunity in vivo [33,34]. Interestingly, anti-ASFV antibodies have been shown to have novel inhibitory effects on ASFV replication in macrophage cell cultures [35]. The continuous presence of convalescent serum (at nearly undiluted to 10% concentrations) inhibited infection of autologous buffy-coat cell cultures with homologous but not heterologous ASFV strains. This monocyte infection-inhibition (M-II) activity was mediated by purified IgG and was effective at inhibiting viral replication after virus adsorption had occurred [36,37]. Most notably, M-II antibody titers in animals appeared to correlate with protection from the challenge [36,37].

Multiple data support a role for cellular immune responses in ASFV protective immunity. Lymphocyte depletion experiments in pigs indicate that cytotoxic CD8+ lymphocytes are important for ASFV clearance and protection [38], and protective effects are correlated with ASFV strain-specific CD8+ T-cell responses [39]. Additionally, IFN-gamma responses in vitro appear to correlate with the degree of cross-protection against heterologous ASFV challenge [21]. Lack of detectable anti-ASFV antibodies at the time of challenge in DNA-vaccinated and partially protected animals has been interpreted as support for the role of cellular immunity in protection [22,39].

Thus, while available data support a role for both humoral and cellular immune responses in protection, definitive immune correlates of protection are lacking [40]. However, the protection afforded by passive transfer of ASFV antibodies discussed above provides compelling evidence for anti-viral antibodies in protective immunity.

### 2.2. ASF Vaccine Approaches

Viral virulence is a relative phenomenon dependent on multiple key variables, including the following: strain of virus, viral dose administered, route of inoculation, and host animal factors. Vaccine protection/efficacy studies are essentially assessments of viral virulence involving pretreatment of the host with a fifth variable, vaccination (using a specific antigenic material/vehicle, dose, route, immunization schedule, and time to challenge). Importantly, protective effects are greatly impacted by vaccination parameters and all variables affecting virulence in a given host. Thus, experimental context is particularly relevant when considering immune protection as presented in current ASF vaccine literature, as studies have been conducted in multiple ways. Tested have been a variety of ASFV strains (European origin, African origin, highly virulent, moderately virulent, unknown virulence, various degrees of tissue culture adaptation, animal passage only, etc.) and variable challenge doses and routes (high LD50, low LD50, sublethal challenge, intramuscular challenge, contact challenge etc.). In addition, variables have been test hosts, including pigs of various ages, breeds, and conditions (conventional, specific-pathogen-free, inbred) widely ranging in age and/or weight, persistently infected with ASFV at time of challenge, etc.). Finally, studies have used a wide range of vaccination protocols/schedules, including variable periods between vaccination and vaccination assessment (animal challenge) and variable standards for protection assessment. Given our limited understanding of ASFV and ASFV infection biology, it is difficult to predict with any degree of certainty the biological significance of these variables and their potential interactions with one another in the context of ASFV infection and protective immunity. As such, it is critical to interpret ASFV virulence and ASF protection results carefully within the context of the specific study and to take care in inferring their generalizability.

### 2.3. Live-Attenuated ASF Viruses (LAVs) as Vaccines

#### 2.3.1. Traditional LAVs as Vaccines

Solid protective immunity is induced in pigs surviving ASFV infection. Pigs infected with moderately virulent ASF viruses or those attenuated by traditional methods develop long-term resistance to homologous, but rarely to heterologous, virus challenge [10,18,19,20,21,22,41]. The boundaries of homologous cross-protection are not always clear, as distinct ASFV may induce measures of cross-protection [21], and conversely, ASFV that appear closely related may fail to cross-protect [22,41]. In general, the ASFV immune protection afforded by ASFV LAVs is characterized by an absence of clinical signs and by a reduction in viremia, which is either absent or delayed in onset and markedly reduced in titer. Together, these are understood to reflect early control of viral replication in the pig.

#### 2.3.2. Engineered LAVs as Vaccines

Theoretically, it should be possible to engineer attenuated ASF viruses with enhanced safety and efficacy profiles over those observed for traditionally generated LAVs. Comparative and functional ASFV genomics research has identified ASFV viral genes associated with viral virulence, host range and immunomodulation (VHRI) [1,2,42,43,44,45]. ASF viruses containing engineered deletions of specific VHRI genes including thymidine kinase (TK), 9GL (B119L), NL (DP71L), DP148R, I177L, 9GL, and multiple members of multigene families 360 and 505 (MGF 360/505) are attenuated in the host and capable of inducing protective immune responses against homologous parental virus challenge [46,47,48,49,50,51,52,53].

Proposed VHRI functions for these ASFV genes likely underlie the attenuated phenotype observed for the gene-deleted viruses in the pig. Attenuation of TK and 9GL gene-deleted ASFV viruses is likely due to the pronounced replication defect observed for these viruses in swine macrophages, the critical target cell for ASFV replication in vivo [47,48]. Deletion of NL from the European ASFV strain E70 reduced its virulence in swine without affecting viral replication and, the NL protein, which shares similarity with the herpes simplex virus ICP34.5 protein, is thought to prevent host-cell protein shutoff by directing dephosphorylation of eIF-2a by protein phosphatase 1a [46]. Suppression of type I IFN responses in the host by ASFV MGF 360/505 genes [54,55] may account for both the macrophage growth defect observed for some MGF 360/505 gene-deleted viruses and their attenuated phenotype in animals [50,52,56].

Effects of gene deletion on ASFV attenuation and immunogenicity may be strain-dependent. For example, deletion of the NL (DP71L) gene completely attenuates the European E70 strain in animals but has no effect in two African ASFV strains [57,58]. Additionally, ASFV strains Malawi and Georgia are both attenuated by TK gene deletion, but only the TK- deleted Malawi virus was capable of inducing a protective immune response in vivo [47,59]. Further, deletion of the CD2-like gene from the moderately virulent European isolate BA71 attenuates the virus, whereas a similar deletion in other virulent African viruses does not [60,61,62].

Multiple attenuating mutations in ASFV may negatively affect viral immunogenicity and protective immunity. When two VHRI genes (NL (DP71L) and U.K. (DP96R)) were deleted from the previously-attenuated ASFV isolate, OUR T88/3 protection in immunized animals was reduced from 100% to 66% [63]. Similarly, while ASFV Georgia recombinant viruses containing single deletions in either 9GL or an MGF 360/505 gene cluster were fully attenuated in pigs and protected them from challenge with virulent ASFV Georgia [49,50], deletion of both the 9GL gene and an MGF 360/505 gene cluster resulted in a highly attenuated virus incapable of inducing protective immune responses in inoculated animals [64].

#### 2.3.3. Safety of ASF LAVs

LAV vaccine safety and efficacy are relative phenomena and must be viewed and evaluated in the context of the same criteria affecting viral virulence, specifically viral isolate, dose of virus administered, route of virus inoculation, and specific characteristics of the inoculated animal. Significant safety issues have been raised for experimental ASF LAVs. Recent ASF outbreaks in China attributed to the use of unlicensed ASF gene-deleted vaccine virus highlight potential problems with the use of ASF LAV vaccines [65].

Post-vaccinal reactions can lead to the development of chronic ASF. This was first observed in pigs vaccinated with a Portuguese ASF isolate attenuated by serial passage in bone marrow cell cultures [5,66]. Similarly, 25–47% of animals inoculated with a naturally occurring attenuated ASFV isolate, ASFV/NH/P68 (likely a vaccine-derived virus [45]) developed chronic lesions and disease characterized by late fever and viremia and by high levels of anti-ASFV antibodies with marked hypergammaglobulinemia [20]. Immunopathologic conditions (including hypergammaglobulinemia and systemic immune activation involving increased numbers of macrophages, activated B-cells, and CD8+ T-cells) were similarly observed in pigs infected with other moderately virulent ASFV isolates [67,68]. Less severe post-vaccinal reactions involving fever and joint swelling were described for a potential ASF LAV vaccine candidate, OUR T88/3 [21]. Recently, it has been suggested that regulatory components of the immune system (regulatory T-cells and IL-10) may inhibit the development of a long duration protective immune response; a failure to generate robust immune responses following vaccination with LAV may underlie the development of chronic disease in some challenged animals [69].

Still other context-dependent safety issues involving viral strain, immunizing dose, route, and host variability also have been reported. Factors involving host immune status and/or co-infection with another pathogen appear to impact ASFV virulence. Examples of ASFV attenuated in conventional pigs, but retaining virulence in specific-pathogen-free pigs, have been described [21,22]. In addition, a safe immunizing dose and route may be a concern. With some attenuated ASFV, the difference between a safe and virulent dose appears to be small and ASFV strain-dependent [22,49].

### 2.4. Inactivated ASF Vaccines

To date, attempts to protect animals from ASF using a variety of traditional inactivated or “killed” vaccines have failed. Examples of ineffective killed ASF vaccine formulations that failed to provide protection include the following: inactivated infected cell extracts, supernatants of infected pig peripheral blood leukocytes, purified and inactivated virions, infected and glutaraldehyde-fixed macrophages, and detergent-treated, infected alveolar macrophages [70,71,72,73]. Recent studies vaccinating with inactivated ASFV and state-of-the-art adjuvants or high doses of the inactivated virus also failed to induce protective responses in vaccinated animals [74,75]. Given available data, the development of efficacious traditional inactivated ASF vaccines appears unlikely.

### 2.5. Subunit ASF Vaccines—Identification of ASFV Protective Antigens

ASF subunit vaccines where only specific protective viral antigens and optimized delivery/vector systems are used to vaccinate the host may improve upon traditional inactivated vaccine approaches and remove safety concerns associated with LAV. Before subunit vaccine strategies can be designed and delivery/vector systems evaluated, relevant ASFV protective antigens (PA) and the breadth of their natural antigenic diversity need to be known. Attempts are being made to identify ASFV PA using a variety of approaches. So far, most studies focused on highly immunogenic ASFV structural proteins, including p30, p54, and p72, delivered to pigs in various combinations as purified recombinant proteins [30,32,76], recombinant DNA [39], or a combination of both [77]. Challenge experiments following immunization with these cocktails have resulted in partial to no significant protection, even though, in some cases, robust immune responses to the proteins were obtained. Additional attempts to identify ASFV immunogenic and potential PA following DNA vaccination, use of adenovirus vectors, immunization with ASFV gene expression libraries, or DNA prime/recombinant vaccinia virus boost approaches also failed to protect challenged pigs from disease [78,79,80,81,82,83,84,85]. Recent studies using a DNA prime/vaccinia virus boost immunization protocol identified a pool of eight ASFV proteins that protected pigs from infection with a virulent Type1 virus OUR T88/1 are promising [86]. It will be interesting to see if similar protection results are obtained using Georgia-07 or more virulent African field isolates.

The ASFV CD2v protein has been implicated in protective immunity. CD2v (also referred to as 8DR or pEP402R) is the only known viral homolog of cellular CD2, a T-cell protein involved in the co-regulation of cell activation. CD2v is the ASFV hemagglutinin necessary and sufficient for mediating hemadsorption by ASFV- infected cells [87,88]. CD2v gene deletion from the virus results in reduced virus replication and generalization of infection in the pig and, the presence of the CD2v protein suppresses cellular immune responses in vitro [89]. Notably, CD2v gene orthologues are among the most divergent between genome sequences of ASFV isolates [1,90], providing antigens of potential significance in serogroup-specific immunity [91].

Pigs immunized with CD2v developed hemadsorption inhibiting (HAI) and monocyte infection-inhibiting (M-II) antibodies that recognized a 75 kDa virion protein and are partially protected from challenge with the homologous virulent virus strain [62,92] expression is required for partial protection conferred by specific vaccine constructs, and two predicted CD2v T-cell epitopes are speculated to affect protective immunity [39,93]. Additional support for CD2v in protective immunity comes from vaccine studies using ASFV chimeric viruses. Here, homologous CD2v (and adjacent C-type lectin protein (EP153R)) were necessary but not sufficient for the induction of protective ASFV immunity [94]. T-cell epitopes were identified and mapped to serogroup-specific (SG) regions of both proteins [95]. Further, it has been shown recently that deletion of CD2-like and C-type lectin-like genes from ASFV Georgia-∆9GL abrogates its effectiveness as a vaccine [96].

Limited studies have indicated that attenuated non-hemadsorbing ASFV viruses (BA71V, OURT88/3, and NHP68, which contain CD2 gene deletion or N-terminal truncating mutations in CD2v/C-type lectin genes and thus presumably lack the proteins) protected pigs and wild boar from virulent virus challenge [20,38,97,98]. These results suggest that other viral proteins also may be important for inducing a protective response and/or alternatively, the experimental design features of these experiments may be responsible.

### 2.6. What Is a Heterologous ASFV Strain?

As discussed above, pigs infected with moderately virulent strains of ASFV, or those attenuated by serial passage, develop long-term resistance to homologous, but rarely to heterologous, virus challenge. In the context of cross-protective immunity, what is a heterologous ASFV strain? Knowledge of ASFV strain diversity and the breadth of strain variation in nature, and notably antigenic diversity of relevant protective antigens, is critical for successful ASFV vaccine design and for the development of rapid diagnostic methods capable of discriminating among viruses and predicting the efficacy of a given vaccine against any ASFV field isolate.

Current ASFV genotyping relies predominantly on the analysis of sequences from a few distinct genetic loci that demonstrate different levels of variability among diverse isolates. In particular, a standard methodology has come to include typing of the p72 capsid protein gene to provide broad inter-genotypic phylogenetic grouping with concurrent analysis of central variable region tandem repeats within the 9RL/B602Land p54/E183L genes or intergenic regions to provide intra-genotypic resolution [90,99,100,101]. To date, greater than 24 ASFV genotypes have been identified. Although useful for some purposes, ASFV genotyping does not fully correlate with available cross-protection data and may be of limited value in predicting cross-protective vaccine efficacy [21,91].

Although ASFV serologic assays used for disease diagnosis have focused on conserved cross-reactive viral proteins [102], evidence indicates distinct antigenic types of ASFV exist based on HAI serologic typing [9,32,103,104,105,106]. Furthermore, HAI typing places ASFV isolates into discrete serogroups (SG) not necessarily resolved by conventional P72 genotyping. For example, ASFV of serogroups 1, 2, and 4 are all p72 genotype I [91]. Eight ASFV HAI serogroups have been identified, although more likely to exist [94,107,108,109,110]. Available data suggest that ASF protective immunity may be SG-specific, as viruses within an SG appear to cross-protect against one another [107,110,111,112].

Recent data demonstrate that CD2v and C-type lectin proteins are necessary and sufficient for mediating HAI serologic specificity and that CD2v/C-type lectin genotyping reliably groups ASFV by SG, facilitating the study of strain diversity [111]. This, combined with the data above implicating CD2v/C-type lectin as ASFV protective antigens, supports the concept of HAI serotype-specific protective immunity. Again, additional viral protective antigens, perhaps also serogroup-associated, likely are involved in solid protective immunity [39,79,94].

## 3. Where We Need to Go

### 3.1. Short Term ASFV Vaccine Priorities

There is a general consensus among the animal health community that the primary ASF research goal should be the development of a safe and efficacious vaccine for ASFV Georgia-07 for immediate use in endemic disease regions. In all likelihood, a first-generation Georgia-07 vaccine will be an LAV. Promising vaccine candidates are available and at various stages of safety and efficacy testing [50,51,53,113]. Optimally, these vaccines also will be differentiate infected from vaccinated-animal (DIVA) compatible and will be suitable for use with a disease control program. These LAV vaccines also would be suitable for oral inoculation [98], thus addressing vaccine needs for wildlife (wild boars, feral pigs, etc.) as well. An effective Georgia-07 vaccine will significantly reduce the risk to swine producers worldwide by combating disease where it is occurring, thus reducing the potential for transmission to nonendemic regions. It also will provide an emergency tool for rapid response and recovery should it be needed following Georgia-07 introduction into a disease-free region.

If potential ASFV vaccines are to move from the laboratory bench to the field, it is imperative that appropriate safety and efficacy testing be performed under field conditions. This will require significant commitment by commercial partners as higher standards of testing, well beyond those traditionally used for animal health vaccines, will be required. The lack of a vaccine market outside of an uncertain and ephemeral one in China and South East Asia, together with the costs of extensive safety/efficacy testing and attendant liability issues, provide strong disincentives for vaccine producers. Increased private and/or governmental funding likely will be necessary to move experimental ASFV vaccines to the field in a timely manner.

Although first-generation ASFV LAV vaccines will positively impact disease control in endemic regions, it is hard to imagine a scenario where they would be used in countries with highly developed swine industries. Here, control will be achieved by quarantine and culling infected and contact animals while maintaining serologically negative national herds. Issues of efficacy, residual pathogenicity with immunopathologic sequelae, and potential for long-term viral persistence raise significant questions about the suitability of current LAV vaccine candidates for use in a nonendemic region.

The real promise for ASF LAV is controlling endemic disease in Africa and the Asia-Pacific regions: an effective vaccine would contribute markedly to food security, economic development, and disease threat reduction for other global regions. Unfortunately, there has been limited progress on the African front and a lack of donor commitment for translating promising laboratory results to practice. The advent of an improved rationally engineered ASF LAV with strong safety profiles may change thinking and energize much-needed action.

### 3.2. Medium- to Longer-Term ASFV Vaccine Priorities

Medium- to longer-term vaccine priorities should be centered on (1) design of second/third generation ASF LAV with broader cross-protection potential and enhanced safety profiles and (2) development of vaccine strategies permitting rapid response to emerging ASFV strains. How do we rapidly adapt a vaccine to meet the threat of a newly emerging ASFV strain? Central to this will be the identification of ASFV PA. Once ASFV PAs are identified, delivery methods/vectors for optimizing host responses can be developed.

#### 3.2.1. Improvements for ASF LAV

Enhanced understanding of ASFV VHRI genes will facilitate rational engineering of second/third generation ASF LAV with broader cross-protection potential and much-enhanced safety profiles. The challenge remains to identify a specific complement of attenuating mutations that function in diverse ASFV genetic backgrounds, maximizing safety without compromising protective immunogenicity. Attenuating mutations may be strain-specific or situational, thus requiring significant effort to identify those that are necessary for attenuation while still maintaining an effective LAV with a strong safety profile. Identification of a common complement of attenuating mutations functioning across multiple viral strains would be invaluable, allowing for the rapid engineering of an LAV for a newly emergent ASFV strain. Given limited research resources, experimental work pursuing an improved second-generation Georgia-07 vaccine should center on using Georgia-07 itself for the identification of VHRI genes and for animal vaccination/challenge experiments. Results using other virus strains may or may not be relevant and may, in fact, provide misleading information toward a safe and effective Georgia-07 vaccine.

Immunopathologic sequelae and persistent infection are of particular concern for LAV vaccine design. As the mechanisms and virus-host interactions underlying ASF LAV immunopathology are not understood, great care needs to be exercised in evaluating potential LAV vaccine candidates for safety. Persistent infection with vaccine virus further complicates vaccine use, raising possibilities of complementing mutations arising during replication of the gene-deleted virus resulting in reversion to virulence. While the significance of persistent ASFV infection for establishing robust, long-lasting protective immunity is unknown, persistence is conceivably a factor that should be considered in LAV vaccine evaluation. Specifically, does protection afforded by ASF LAVs involve viral interference mechanisms that result from a persistent viral infection, or does viral persistence in some way potentiate adaptive protective immune mechanisms? An improved understanding of ASFV infection biology and viral VHRI genes should lead to progress in addressing both of these critical issues.

Progress on functional ASFV genomics also may allow for the rational design of host range restricted ASF LAV (HRR-LAV) or ASFV-based expression vectors (ASFV-EV). Research investment in the design/development of a well-characterized ASFV-EV with an enhanced safety and efficacy profile, and perhaps HRR, is warranted as a tool to rapidly respond to newly emerging ASFV strains. Here, critical PA from a newly emerging ASFV strain could be engineered into the well-characterized ASFV-EV backbone vector, rapidly creating an LAV vaccine for the newly emerging virus strain. An ASFV-EV DIVA compatible vaccine offers potential benefits: it ensures proper expression levels of PAs (native promoter used and expressed appropriately during ASFV replication), authentic post-translational modifications of the putative ASFV PA in the context of the ASFV infected cell, delivery of the appropriate antigen dose, and proper antigen presentation to the host in the context of natural ASFV infection.

An ASF-HRR vaccine (incapable of replication in either host-pig, tick, or both) would have a markedly enhanced safety profile, and it may be possible to engineer a virus incapable of infecting both pigs and ticks. A tick-HRR vaccine would break the cycle of transmission in nature, preventing the vaccine virus from establishing itself in the tick host, where selective pressures may lead to reversion to virulence and/or recombination events with other viruses [114,115,116]. However, generating robust protective responses with an HRR vaccine may be problematic should they generate light antigen loads and limit antigen presentation to the host.

#### 3.2.2. Subunit ASF Vaccines: Identification of ASFV Protective Antigen (PA)

Perhaps the single greatest ASFV research challenge is the identification of PA. ASF subunit vaccines where only specific protective viral antigens and optimized delivery/vector systems are used to vaccinate the host will improve upon traditional inactivated vaccine approaches and remove safety concerns associated with LAV. Before subunit vaccine strategies can be designed and delivery/vector systems evaluated, relevant ASFV PA and the breadth of their natural antigenic diversity need to be known. Identification of strong correlates of protective immunity, which have proved elusive to date, should emerge in the context of PA-specific host responses.

Although several ASFV proteins have been associated with protection, no specific viral protein(s) alone have been shown sufficient for the induction of robust protective immunity in pigs. This failure likely indicates that responses to multiple viral antigens or antigens yet to be identified are required for solid protection. Alternatively, improved immunization strategies including proper expression and post-translational modification of the putative ASFV protective antigen(s), delivery of the appropriate antigen dose, and proper antigen presentation to the host in a context mimicking viral infection may be necessary to markedly enhance the protection results obtained using specific ASFV proteins. To date, most of the experimental approaches used to identify ASFV PA have involved using two unknowns to study each other; ASFV protective antigens are not known, nor are ways to optimally present ASFV antigens to the host to induce protective host responses. Research creativity and the ability to conduct large-scale vaccination/challenge experiments in pigs will be necessary for timely progress in this area.

Once ASFV PA are identified, it should be possible to evaluate delivery methods/vectors for optimal antigen delivery. A subunit or vector-based antigen cassette strategy will permit rapid adaptation of a well-characterized vaccine platform for newly emerging ASF viruses. For example, an ASFV-EV (already thoroughly evaluated for safety and efficacy) containing PA of the new ASFV strain could be constructed rapidly and used with limited safety and efficacy testing required.

In addition, knowledge of relevant ASF PA will enable the development of rapid genotyping methods capable of discriminating among viruses and predicting the efficacy of a given vaccine for a newly identified field isolate. A better understanding of diversity among PA will also facilitate the design and development of multivalent antigens capable of immunizing animals against multiple viral strains; this capability would be invaluable where significant strain diversity is present within a geographic region.

There is an outside possibility that subunit/vectored vaccines may never achieve the efficacy needed for full protection from ASFV. Notably, all potential ASV vaccines described to date have been LAV, and protection results following immunization with individual or a subset of ASFV proteins have proven disappointing. It is possible that subunit/vectored vaccines may not induce the qualitative and/or quantitative level of protective responses observed for LAV. Furthermore, the significance of persistent ASFV infection and its contribution to achieving robust protective immunity is unknown. In this scenario, an ASFV-EV-based vaccine may prove particularly useful for inducing necessary protective responses.

### 3.3. Strain Diversity

ASFV HAI serogrouping and CD2v/C-type lectin genotyping appear to provide a new paradigm to examine aspects of ASF strain variation in the context of cross-protective immunity. Further work will be needed to determine their robustness and the genetic and antigenic bounds of cross-protective immunity. With cost-effective rapid sequencing technologies now available, better genetic characterization of ASFV diversity in sub-Saharan Africa is possible. Genetic characterization of ASFV strains combined with traditional cross-protection experiments in pigs should provide insight for placing viruses into cross-protective groups. Comparative sequence analysis of ASFV PA may simplify the process allowing for rapid assignment of ASFV strains to cross-protective groups.

## 4. Summary

There is consensus among the animal health community that the primary ASF research goal should be the development of a safe and efficacious vaccine for ASFV Georgia-07 for immediate use in endemic disease regions. In all likelihood, a first-generation Georgia-07 vaccine will be an LAV. Appropriate safety and efficacy testing under field conditions will be necessary to move vaccine candidates from the laboratory bench to the field. Enhanced understanding of VHRI genes will facilitate rational engineering of second/third generation ASF LAV with broader cross-protection potential and much-enhanced safety profiles. An effective Georgia-07 vaccine will significantly reduce the risk to swine producers worldwide by combating disease where it is currently occurring, thus reducing the potential for transmission to disease-free regions. It also will provide an emergency tool for rapid response and recovery should it be needed following Georgia-07 introduction into disease-free regions. Medium- to longer-term ASFV vaccine priorities should be centered on the development of vaccine strategies permitting rapid response to emerging ASFV strains. Central to this will be the identification of ASFV PA. Once ASFV PA are identified, delivery methods/vectors for optimizing host responses can be developed. A subunit or vector-based antigen cassette strategy will permit rapid adaptation of a well-characterized vaccine delivery platform for newly emerging ASF viruses.

## Data Availability

Not applicable.

## References

[1] Tulman E.R., Delhon G., Ku B., Rock D.L., Van Etten J. (2009). Asfarviruses. Lesser Known Big DNA Viruses-Current Topics in Microbiology and Immunology.

[2] Chapman D.A.G., Tcherepanov V., Upton C., Dixon L.K. (2008). Comparison of the genome sequences of non-pathogenic and pathogenic African swine fever virus isolates. J. Gen. Virol..

[3] Villeda C.J., Williams S.M., Wilkinson P.J., Vinuela E. (1993). Consumption coagulopathy associated with shock in acute ASF. Arch. Virol..

[4] Villeda C.J., Gomez-Villamandos C., Williams S.M., Hervas J., Wilkinson P.J., Vinuela E. (1995). The role of fibrinolysis in the pathogenesis of the haemorrhagic syndrome produced by virulent isolates of ASFV. Thromb. Haemost..

[5] Moulton J., Coggins L. (1968). Comparison of lesions in acute and chronic African swine fever. Cornell Vet..

[6] Colgrove G.S., Haelterman E.O., Coggins L. (1969). Pathogenesis of African swine fever in young pigs. Am. J. Vet. Res..

[7] Konno S., Taylor W.D., Dardiri A.H. (1971). Acute African swine fever proliferative phase in lymphoreticular tissue and the reticuloendothelial system. Cornell Vet..

[8] DeKock G., Robinson E.M., Keppel J.J.G. (1940). Swine fever in south-Africa. Onderstepoort J. Vet. Sci. Anim. Ind..

[9] Detray D.E. (1957). Persistence of viremia and immunity in African swine fever. Am. J. Vet. Res..

[10] Mebus C.A., Dardiri A.H. (1980). Western hemisphere isolates of African swine fever virus: Asymptomatic carriers and resistance to challenge inoculation. Am. J. Vet. Res..

[11] Wilkinson P.J., Wardley R.C., Williams S.M., Wilkinson P.J. (1983). Studies in pigs infected with African swine fever virus. African Swine Fever, Proceedings of a FAO/CEC Research Seminar Held in Sassari, Sardinia, 23–25 September 1981.

[12] De Carvalho Ferreira H.C., Weesendorp E., Elbers A.R., Bouma A., Quak S., Stegeman J.A., Loeffen W.L. (2012). African swine fever virus excretion patterns in persistently infected animals: A quantitative approach. Vet. Microbiol..

[13] Carrillo C., Borca M.V., Afonso C.L., Onisk D.V., Rock D.L. (1994). Longterm persistent infection of swine monocytes/macrophages with African swine fever virus. J. Virol..

[14] Stahl K., Sternberg-Lewerin S., Blome S., Viltrop A., Penrith Mary-Louise Chenais E. (2019). Lack of long term carriers of African swine fever virus—A systematic review. Virus Res..

[15] Thomas L.F., Bishop R.P., Onzere C., Mcintosh M.T., Lemire K.A., de Glanville W.A., Cook E.A.J., Fevre E.M. (2016). Evidence for the persistence of African swine fever virus in an endemic region of Western Kenya in the absence of any reported outbreak. BMC Vet. Res..

[16] Chambaro H.M., Sasaki M., Sinkala Y., Gonzalez G., Squarre D., Fandamu P., Lubaba C., Mataa L., Shawa M., Mwape K.E. (2020). Evidence for exposure of asymptomatic domestic pigs to African swine fever virus during an inter-epidemic period in Zambia. Transbound Emerg. Dis..

[17] Montgomery R.E. (1921). On a form of swine fever occurring in British East Africa (Kenya colony). J. Comp. Pathol..

[18] Ruiz-Gonzalvo F., Carnero E., Bruyel V., Wilkinson P.J. (1983). Immunological responses of pigs to partially attenuated African swine fever virus and their resistance to virulent homologous and heterologous viruses. African Swine Fever. Proc. EUR 8466 EN.

[19] Hamdy F.M., Dardiri A.H. (1984). Clinic and immunologic responses of pigs to African swine virus isolated from the Western hemisphere. Am. J. Vet. Res..

[20] Leitão A., Cartaxeiro C., Coelho R., Cruz B., Parkhouse R.M., Portugal F., Vigário J.D., Martins C.L. (2001). The non-haemadsorbing African swine fever virus isolate ASFV/NH/P68 provides a model for defining the protective anti-virus immune response. J. Gen. Virol..

[21] King K., Chapman D., Argilaguet J.M., Fishbourne E., Hutet E., Cariolet R., Hutchings G., Oura C.A., Netherton C.L., Moffat K. (2011). Protection of European domestic pigs from virulent African isolates of African swine fever virus by experimental immunization. Vaccine.

[22] Lacasta A., Monteagudo P.L., Jiménez-Marín Á., Accensi F., Ballester M., Argilaguet J., Galindo-Cardiel I., Segalés J., Salas M.L., Domínguez J. (2015). Live attenuated African swine fever viruses as ideal tools to dissect the mechanisms involved in viral pathogenesis and immune protection. Vet. Res..

[23] Schlafer D.H., Mebus C.A., McVicar J.W. (1984). African swine fever convalescent sow: Subsequent pregnancy and effect of colostral antibody on challenge inoculation of their pigs. Am. J. Vet. Res..

[24] Schlafer D.H., Mebus C.A., McVicar J.W. (1984). African swine fever in neonatal pigs: Passively acquired protection from colostrum or serum from recovered pigs. Am. J. Vet. Res..

[25] Onisk D.V., Borca M.V., Kutish G.F., Kramer E., Irusta P., Rock D.L. (1994). Passively transferred African swine fever virus antibodies protect swine against lethal infection. Virology.

[26] Ruiz-Gonzalvo F., Caballero C., Martinez J., Carnero M.E. (1986). Neutralization of African swine fever virus by sera from African swine fever-resistant pigs. Am. J. Vet. Res..

[27] Zsak L., Onisk D.V., Afonso C.L., Rock D.L. (1993). Virulent African swine fever virus isolates are neutralized by swine immune serum and by monoclonal antibodies recognizing a 72-kDa viral protein. Virology.

[28] Borca M.V., Irusta P., Carrillo C., Afonso C.L., Burrage T., Rock D.L. (1994). African swine fever virus structural protein p72 contains a conformational neutralizing epitope. Virology.

[29] Gómez-Puertas P., Rodríguez F., Oviedo J.M., Ramiro-Ibáñez F., Ruiz-Gonzalvo F., Alonso C., Escribano J.M. (1996). Neutralizing antibodies to different proteins of African swine fever virus inhibit both virus attachment and internalization. J. Virol..

[30] Gómez-Puertas P., Rodríguez F., Oviedo J.M., Brun A., Alonso C., Escribano J.M. (1998). The African swine fever virus proteins p54 and p30 are involved in two distinct steps of virus attachment and both contribute to the antibody-mediated protective immune response. Virology.

[31] Escribano J.M., Galindo I., Alonso C. (2013). Antibody-mediated neutralization of African swine fever virus:myths and facts. Virus Res..

[32] Neilan J.G., Zsak L., Lu Z., Burrage T.G., Kutish G.F., Rock D.L. (2004). Neutralizing antibodies to African swine fever virus proteins p30, p54, and p72 are not sufficient for antibody-mediated protection. Virology.

[33] Norley S.G., Wardley R.C. (1982). Complement-mediated lysis of African swine fever virus-infected cells. Immunology.

[34] Norley S.G., Wardley R.C. (1983). Effector mechanisms in the pig: Antibody- dependent cellular cytolysis of African swine fever virus infected cells. Res. Vet. Sci..

[35] Malmquist W. (1963). Serologic and immunologic studies with African swine fever virus. Am. J. Vet. Res..

[36] Ruiz-Gonzalvo F., Carnero M.E., Caballero C., Martinez J. (1986). Inhibition of African swine fever infection in the presence of immune sera in vivo and in vitro. Am. J. Vet. Res..

[37] Knudsen R.C., Genovesi E.V., Whyard T.C. (1987). In vitro immune serum-mediated protection of pig monocytes against African swine fever virus. Am. J. Vet. Res..

[38] Oura C.A., Denyer M.S., Takamatsu H., Parkhouse R.M. (2005). In vivo depletion of CD8+ T lymphocytes abrogates protective immunity to African swine fever virus. J. Gen. Virol..

[39] Argilaguet J.M., Pérez-Martín E., Nofrarías M., Gallardo C., Accensi F., Lacasta A., Mora M., Ballester M., Galindo-Cardiel I., López-Soria S. (2012). DNA vaccination partially protects against African swine fever virus lethal challenge in the absence of antibodies. PLoS ONE.

[40] Carlson J., O’Donnell V., Alfano M., Velazquez Salinas L., Holinka L.G., Krug P.W., Gladue D.P., Higgs S., Borca M.V. (2016). Association of the Host Immune Response with Protection Using a Live Attenuated African Swine Fever Virus Model. Viruses.

[41] Mulumba-Mfumu L.K., Goatley L.C., Saegerman C., Takamatsu H.H., Dixon L.K. (2015). Immunization of African indigenous pigs with Attenuated genotype I African swine fever virus OURT88/3 induces protection against challenge with virulent strains of genotype I. Transbound. Emerg. Dis..

[42] Tulman E.R., Rock D.L. (2001). Novel Virulence and host range genes of African swine fever virus. Curr. Opin. Microbiol..

[43] Dixon L.K., Abrams C.C., Bowick G., Goatley L.C., Kay-Jackson P.C., Chapman D., Liverani E., Nix R., Silk R., Zhang F. (2004). African swine fever virus proteins involved in evading host defense systems. Vet. Immunol. Immunopathol..

[44] Corria S., Ventura S., Parkhouse R.M. (2013). Identification and utility of innate immune system evasion mechanism of ASFV. Virus Res..

[45] Portugal R., Coelho J., Hoper D., Little N.S., Smithson C., Upton C., Martins C., Leitao A., Keil G.M. (2015). Related strains of African swine fever virus with different virulence: Genome comparison and analysis. J. Gen. Virol..

[46] Zsak L., Lu Z., Kutish G.F., Neilan J.G., Rock D.L. (1996). An African swine fever virus virulence-associated gene NL-S with similarity to the herpes simplex virus ICP34. 5 gene. J. Virol..

[47] Moore D.M., Zsak L., Neilan J.G., Lu Z., Rock D.L. (1998). The African swine fever virus thymidine kinase gene is required for efficient replication in swine macrophages and for virulence in swine. J. Virol..

[48] Lewis T., Zsak L., Burrage T.G., Lu Z., Kutish G.F., Neilan J.G., Rock D.L. (2000). An African swine fever virus ERV1-ALR homologue, 9GL, affects virion maturation and viral growth in macrophages and viral virulence in swine. J. Virol..

[49] O’Donnell V., Holinka L.G., Krug P.W., Gladue D.P., Carlson J., Sanford B., Alfano M., Kramer E., Lu Z., Arzt J. (2015). African swine fever virus Georgia 2007 with a deletion of virulence-associated gene 9GL (B119L), when administered at low doses, leads to virus attenuation in swine and induces an effective protection against homologous challenge. J. Virol..

[50] O’Donnell V., Holinka L.G., Gladue D.P., Sanford B., Krug P.W., Lu X., Arzt J., Reese B., Carrillo C., Risatti G.R. (2015). African swine fever virus Georgia isolate harboring deletions of MGF360 and MGF505 genes is attenuated in swine and confers protection against challenge with the virulent parental virus. J. Virol..

[51] O’Donnell V., Risatti G.R., Holinka L.G., Krug P.W., Carlson J., Velazquez-Salinas L., Azzinaro P.A., Gladue D.P., Borca M.V. (2016). Simultaneous Deletion of the 9GL and UK Genes from the African Swine Fever Virus Georgia 2007 Isolate Offers Increased Safety and Protection against Homologous Challenge. J. Virol..

[52] Reis A.L., Goatley L.C., Jabbar T., Sanchez-Cordon P.J., Netherton C.L., Chapman D.A.G., Dixon L.K. (2017). Deletion of the African Swine Fever Virus Gene DP148R Does Not Reduce Virus Replication in Culture but Reduces Virus Virulence in Pigs and Induces High Levels of Protection against Challenge. J. Virol..

[53] Borca M.V., Ramirez-Medina E., Silva E., Vuono E., Rai A., Pruitt S., Holinka L.G., Velazquez-Salinas L., Zhu J., Gladue D.P. (2020). Development of a Highly Effective African Swine Fever Virus Vaccine by Deletion of the I177L Gene Results in Sterile Immunity against the Current Epidemic Eurasia Strain. J. Virol..

[54] Afonso C.L., Piccone M.E., Zaffuto K.M., Neilan J.G., Kutish G.F., Lu Z., Balinsky C.A., Gibb T.R., Bean T.J., Zsak L. (2004). African swine fever virus multigene family 360 and 530 genes affect host interferon response. J. Virol..

[55] Golding J.P., Goatley L., Goodbourn S., Dixon L.K., Taylor G., Netherton C.L. (2016). Sensitivity of African swine fever virus to type I interferon is linked to genes within multigene families 360 and 505. Virology.

[56] Zsak L., Lu Z., Burrage T.G., Neilan J.G., Kutish G.F., Moore D.M., Rock D.L. (2001). African swine fever virus multigene family 360 and 530 genes are novel macrophage host range determinants. J. Virol..

[57] Afonso C.L., Zsak L., Carrillo C., Borca M.V., Rock D.L. (1998). African swine fever virus NL gene is not required for virus virulence. J. Gen. Virol..

[58] Neilan J.G., Zsak L., Lu Z., Kutish G.F., Afonso C.L., Rock D.L. (2002). A novel swine virulence determinant in the left variable region of the African swine fever virus genome. J. Virol..

[59] Sanford B., Holinka L.G., O’Donnell V., Krug P.W., Carlson J., Alfano M., Carrillo C., Wu P., Lowe A., Risatti G.R. (2016). Deletion of the thymidine kinase gene induces complete attenuation of the Georgia isolate of African swine fever virus. Virus Res..

[60] Monteagudo P.L., Lacasta A., López E., Bosch L., Collado J., Pina-Pedrero S., Correa-Fiz F., Accensi F., Navas M.J., Vidal E. (2017). BA71ΔCD2: A New Recombinant Live Attenuated African Swine Fever Virus with Cross-Protective Capabilities. J. Virol..

[61] Borca M.V., O’Donnell V., Holinka L.G., Risatti G.R., Ramirez-Medina E., Vuono E.A., Shi J., Pruitt S., Rai A., Silva E. (2020). Deletion of CD2-like gene from the genome of African swine fever virus strain Georgia does not attenuate virulence in swine. Sci. Rep..

[62] Ruız-Gonzalvo F., Rodrıguez F., Escribano J.M. (1996). Functional and immunological properties of the baculovirus expressed hemagglutinin of African swine fever virus. Virology.

[63] Abrams C.C., Goatley L., Fishbourne E., Chapman D., Cooke L., Oura C.A., Netherton C.L., Takamatsu H.H., Dixon L.K. (2013). Deletion of virulence- associated genes from attenuated African swine fever virus isolate OUR T88/3 decreases its ability to protect against challenge with virulent virus. Virology.

[64] O’Donnell V., Holinka L.G., Sanford B., Krug P.W., Carlson J., Pacheco J.M., Reese B., Risatti G.R., Gladue D.P., Borca M.V. (2016). African swine fever virus Georgia isolate harboring deletions of 9GL and MGF360/505 genes is highly attenuated in swine but does not confer protection against parental virus challenge. Virus Res..

[65] Reuters 1-21-21 New China Swine Fever Strains Point to Unlicensed Vaccines. https://www.reuters.com/article/us-china-swinefever-vaccines-insightidUSKBN29R00X.

[66] Manso-Ribeiro J., Nunes-Petisca J.L., Lopez-Frazao F., Sobral M. (1963). Vaccination against ASF. Bull. Off. Int. Epizoot..

[67] Pan I.C., DeBoer C.J., Heuschele W.P. (1970). Hypergammaglobulinemia in swine infected with African swine fever virus. Proc. Soc. Exp. Biol. Med..

[68] Takamatsu H., Denyer M.S., Oura C., Childerstone A., Andersen J.K., Pullen L., Parkhouse R.M. (1999). African swine fever virus: A B cell-mitogenic virus in vivo and in vitro. J. Gen. Virol..

[69] Sánchez-Cordón P.J., Jabbar T., Chapman D., Dixon L.K., Montoya M. (2020). Absence of Long-Term Protection in Domestic Pigs Immunized with Attenuated African Swine Fever Virus Isolate OURT88/3 or BeninΔMGF Correlates with Increased Levels of Regulatory T Cells and Interleukin-10. J. Virol..

[70] Stone S.S., Hess W.R. (1967). Antibody response to inactivated preparations of African swine fever virus in pigs. Am. J. Vet. Res..

[71] Forman A.J., Wardley R.C., Wilkinson P.J. (1982). The immunological response of pigs and guinea pigs to antigens of African swine fever virus. Arch. Virol..

[72] Kihm U., Ackerman M., Mueller H., Pool R., Becker Y. (1987). Approaches to vaccination. African Swine Fever.

[73] Mebus C.A. (1988). African swine fever. Adv. Virus Res..

[74] Blome S., Gabriel C., Beer M. (2014). Modern adjuvants do not enhance the efficacy of an inactivated African swine fever virus vaccine preparation. Vaccine.

[75] Cadenas-Fernández E., Sánchez-Vizcaíno J.M., van den Born E., Kosowska A., van Kilsdonk E., Fernández-Pacheco P., Gallardo C., Arias M., Barasona J.A. (2021). High Doses of Inactivated African Swine Fever Virus Are Safe, but Do Not Confer Protection against a Virulent Challenge. Vaccines.

[76] Barderas M.G., Rodríguez F., Gómez-Puertas P., Avilés M., Beitia F., Alonso C., Escribano J.M. (2001). Antigenic and immunogenic properties of a chimera of two immunodominant African swine fever virus proteins. Arch. Virol..

[77] Sunwoo S.Y., Pérez-Núñez D., Morozov I., Sánchez E.G., Gaudreault N.N., Trujillo J.D., Mur L., Nogal M., Madden D., Urbaniak K. (2019). DNA-Protein Vaccination Strategy Does Not Protect from Challenge with African Swine Fever Virus Armenia 2007 Strain. Vaccines.

[78] Lacasta A., Ballester M., Monteagudo P.L., Rodriguez J.M., Salas M.L., Accensi F., Pina-Pedrero S., Bensaid A., Argilaguet J., Lopez-Soria S. (2014). Expression library immunization can confer protection against lethal challenge with African swine fever virus. J. Virol..

[79] Lopera-Madrid J., Osorio J.E., He Y., Xiang Z., Adams L.G., Laughlin R.C., Mwangi W., Subramanya S., Neilan J., Brake D. (2017). Safety and immunogenicity of mammalian cell derived and Modified Vaccinia Ankara vectored African swine fever subunit antigens in swine. Vet. Immunol. Immunopathol..

[80] Jancovich J.K., Chapman D., Hansen D.T., Robida M.D., Loskutov A., Craciunescu F., Borovkov A., Kibler K., Goatley L., King K. (2018). Immunization of Pigs by DNA Prime and Recombinant Vaccinia Virus Boost To Identify and Rank African Swine Fever Virus Immunogenic and Protective Proteins. J. Virol..

[81] Natasha N.G., Richt J.A. (2019). Subunit vaccine approaches for African Swine Fever Virus. Vaccines.

[82] Lokhandwala S., Petrovan V., Popescu L., Sangewar N., Elijah C., Stoian A., Olcha M., Ennen L., Bray J., Bishop R.P. (2019). Adenovirus-vectored African Swine Fever Virus antigen cocktails are immunogenic but not protective against intranasal challenge with Georgia 2007/1 isolate. Vet. Microbiol..

[83] Murgia M.V., Mogler M., Certoma A., Green D., Monaghan P., Williams D.T., Rowland R.R.R., Gaudreault N.N. (2019). Evaluation of an African swine fever (ASF) vaccine strategy incorporating priming with an alphavirus-expressed antigen followed by boosting with attenuated ASF virus. Arch. Virol..

[84] Netherton C.L., Goatley L.C., Reis A.L., Portugal R., Nash R.H., Morgan S.B., Gault L., Nieto R., Norlin V., Gallardo C. (2019). Identification and Immunogenicity of African Swine Fever Virus Antigens. Front. Immunol..

[85] Cadenas-Fernández E., Sánchez-Vizcaíno J.M., Kosowska A., Rivera B., Mayoral-Alegre F., Rodríguez-Bertos A., Yao J., Bray J., Lokhandwala S., Mwangi W. (2020). Adenovirus-vectored African Swine Fever Virus Antigens Cocktail Is Not Protective against Virulent Arm07 Isolate in Eurasian Wild Boar. Pathogens.

[86] Goatley L.C., Reis A.L., Portugal R., Goldswain H., Shimmon G.L., Hargreaves Z., Ho C.S., Montoya M., Sánchez-Cordón P.J., Taylor G. (2020). A Pool of Eight Virally Vectored African Swine Fever Antigens Protect Pigs Against Fatal Disease. Vaccines.

[87] Rodríguez J.M., Yáñez R.J., Almazán F., Viñuela E., Rodriguez J.F. (1993). African swine fever virus encodes a CD2 homolog responsible for the adhesion of erythrocytes to infected cells. J. Virol..

[88] Borca M.V., Kutish G.F., Afonso C.L., Irusta P., Carrillo C., Brun A., Sussman M.D., Rock D.L. (1994). An African swine fever virus gene with similarity to the T- lymphocyte surface antigen CD2 mediates hemadsorption. Virology.

[89] Borca M.V., Carrillo C., Zsak L., Laegreid W.W., Kutish G.F., Neilan J.G., Burrage T.G., Rock D.L. (1998). Deletion of a CD2-like gene, 8-DR, from African swine fever virus affects viral infection in domestic swine. J. Virol..

[90] Gallardo C., Mwaengo D.M., Macharia J.M., Arias M., Taracha E.A., Soler A., Okoth E., Martín E., Kasiti J., Bishop R.P. (2009). Enhanced discrimination of African swine fever virus isolates through nucleotide sequencing of the p54, p72, and pB602L (CVR) genes. Virus Genes.

[91] Malogolovkin A., Burmakina G., Titov I., Sereda A., Gogin A., Baryshnikova E., Kolbasov D. (2015). Comparative analysis of African swine fever virus genotypes and serogroups. Emerg. Infect. Dis..

[92] Ruiz-Gonzalvo F., Coll J.M. (1993). Characterization of a soluble hemagglutinin induced in African swine fever virus-infected cells. Virology.

[93] Argilaguet J.M., Pérez-Martín E., López S., Goethe M., Escribano J.M., Giesow K., Keil G.M., Rodríguez F. (2013). BacMam immunization partially protects pigs against sublethal challenge with African swine fever virus. Antivir. Res..

[94] Burmakina G., Malogolovkin A., Tulman E.R., Zsak L., Delhon G., Diel D.G., Shobogorov N., Morgunov Y., Morgunov S., Kutish G.F. (2016). African swine fever virus serotype-specific proteins are significant protective antigens for African swine fever. J. Gen. Virol..

[95] Burmakina G., Malogolovkin A., Tulman E., Delhon G., Kolbasov D., Rock D.L. (2019). Identification of T-Cell epitopes in African swine fever virus CD2v and C-type lectin proteins. J. Gen. Virol..

[96] Gladue D.P., O’Donnell V., Ramirez-Medina E., Rai A., Pruitt S., Vuono E.A., Silva E., Velazquez-Salinas L., Borca M.V. (2020). Deletion of CD2-Like (CD2v) and C-Type Lectin-Like (EP153R) Genes from African Swine Fever Virus Georgia-∆9GL Abrogates Its Effectiveness as an Experimental Vaccine. Viruses.

[97] Boinas F.S., Hutchings G.H., Dixon L.K., Wilkinson P.J. (2004). Characterization of pathogenic and non-pathogenic African swine fever virus isolates from Ornithodoros erraticus inhabiting pig premises in Portugal. J. Gen. Virol..

[98] Barasona J.A., Gallardo C., Cadenas-Fernández E., Jurado C., Belén R., Rodríguez-Bertos A., Arias M., Sánchez-Vizcaíno J.M. (2019). First Oral Vaccination of Eurasian Wild Boar against African Swine Fever Virus Genotype II. Front. Vet. Sci..

[99] Bastos A.D., Penrith M.L., Crucière C., Edrich J.L., Hutchings G., Roger F., Couacy-Hymann E.R., Thomson G. (2003). Genotyping field strains of African swine fever virus by partial p72 gene characterization. Arch. Virol..

[100] Lubisi B.A., Bastos A.D., Dwarka R.M., Vosloo W. (2007). Intra-genotypic resolution of African swine fever viruses from an East African domestic pig cycle: A combined p72-CVR approach. Virus Genes.

[101] Nix R.J., Gallardo C., Hutchings G., Blanco E., Dixon L.K. (2006). Molecular epidemiology of African swine fever virus studied by analysis of four variable genome regions. Arch. Virol..

[102] Cubillos C., Gómez-Sebastian S., Moreno N., Nuñez M.C., Mulumba-Mfumu L.K., Quembo C.J., Heath L., Etter E.M., Jori F., Escribano J.M. (2013). African swine fever virus serodiagnosis: A general review with a focus on the analyses of African serum samples. Virus Res..

[103] Vigario J.D., Terrinha A.M., Bastos A.L., Moura-Nunes J.F., Marques D., Silva J.F. (1970). Serological behavior of isolated African swine fever virus. Arch. Gesamte Virusforsch..

[104] Vigario J.D., Terrinha A.M., Moura Nunes J.F. (1974). Antigenic relationships among strains of African swine fever virus. Arch. Gesamte Virusforsch..

[105] Pan I.C., Trautman R., Hess W.R., DeBoer C.J., Tessler J., Ordas A., Botija C.S., Ovejero J., Sanchez M.C. (1974). African swine fever: Comparison of four serotests on porcine serums in Spain. Am. J. Vet. Res..

[106] Coggins L. (1968). A modified hemadsorption-inhibition test for African swine fever virus. Bull. Epizoot. Dis. Afr..

[107] Vishnjakov I.F., Mitin N.I., Petrov J.I. (1995). Seroimmunological classification of African swine fever virus natural isolates. Topical Issues of Veterinary Virology. Proceedings of the Conference VNIIVViM: Classical Swine Fever Urgent Problems of Science and Practice.

[108] Vishnjakov I., Mitin N., Karpov G., Kurinnov V., Jashin A. (1991). Differentiation African and classical swine fever viruses. Veterinariya.

[109] Sereda A.D., Solovkin S.L., Fugina L.G., Makarov V.V. (1992). Immune reactions to the African swine fever virus. Vopr. Virusol..

[110] Balyshev V.M., Fedorishhev I.V., Salina M.V. (1995). Study of serotype interactions of ASF virus strains both in vitro and in vivo. Viral Diseases of Animals.

[111] Malogolovkin A., Burmakina G., Tulman E.R., Delhon G., Diel D.G., Salnikov N., Kutish G.F., Kolbasov D., Rock D.L. (2015). African swine fever virus CD2 v and C-type lectin gene loci mediate serological specificity. J. Gen. Virol..

[112] Sereda A.E., Balyshev V.M., Kazakova A.S., Imatdinov A.R., Kolbasov D.V. (2020). Protective properties of attenuated strains of African swine fever virus belonging to seroimmunotypes I–VIII. Pathogens.

[113] Chen W., Zhao D., He X., Liu R., Wang Z., Zhang X., Li F., Shan D., Chen H., Zhang J. (2020). A seven-gene-deleted African swine fever virus is safe and effective as a live attenuated vaccine in pigs. Sci. China Life Sci..

[114] Kleiboeker S.B., Burrage T.G., Scoles G.A., Fish D., Rock D.L. (1998). African swine fever virus infection in the argasid host, *Ornithodoros porcinus porcinus*. J. Virol..

[115] Kleiboeker S.B., Scoles G.A., Burrage T.G., Sur J. (1999). African swine fever virus repli- cation in the midgut epithelium is required for infection of ornithodoros-ticks. J. Virol..

[116] Rowlands R.J., Duarte M.M., Boinas F., Huychings G., Dixon L.K. (2009). The CD2v protein enhances African swine fever virus replication in the tick vector, *Ornithodoros erraticus*. Virology.

